# Varicella Zoster Virus Vasculopathy: An Under-Recognized Entity

**DOI:** 10.7759/cureus.61419

**Published:** 2024-05-31

**Authors:** Anil M Philip, Lina J George, Anas N, Jemimah Nayar

**Affiliations:** 1 Internal Medicine, Kuriakose Chavara Memorial Hospital, Nooranad, IND; 2 Pulmonology, Kuriakose Chavara Memorial Hospital, Nooranad, IND; 3 Radiology, Metro Scans and Diagnostic Centre, Karunagappalli, IND; 4 Nuclear Medicine, St. Gregorios International Cancer Care Centre, Parumala, IND

**Keywords:** varicella-zoster virus, herpes zoster ophthalmicus, cerebrovascular accident (stroke), varicella zoster vasculopathy, vzv encephalitis

## Abstract

Varicella zoster virus (VZV) vasculopathy is a rare yet potentially severe neurological manifestation resulting from VZV reactivation, primarily affecting immunocompromised individuals.

We present a case report of a 61-year-old male with VZV vasculopathy who initially presented with herpes zoster ophthalmicus, subsequently complicated by meningoencephalitis and an acute infarct in the territory of the left middle cerebral artery (MCA). Imaging revealed acute and chronic infarcts in the capsuloganglionic regions, accompanied by thickening and enhancement of the left MCA wall. Treatment involved a 14-day course of intravenous acyclovir, supplemented with oral prednisolone, resulting in modest clinical improvement.

VZV vasculopathy represents an infrequently acknowledged neurological syndrome, particularly prevalent among immunocompromised individuals. Early recognition and appropriate intervention offer promise in ameliorating outcomes for affected patients. This case emphasizes the importance of including VZV vasculopathy in the differential diagnosis of neurological deficits, especially within high-risk populations.

## Introduction

Before the COVID-19 pandemic, it was well established that the varicella-zoster virus (VZV) stood as one of the primary culprits behind virus-induced central nervous system vasculopathy [1]. VZV, an alpha herpes virus, initiates its course with a primary infection characterized by a disseminated rash known as Varicella or chickenpox. Subsequently, it lies dormant within the ganglia spanning the entire neuraxis. In the elderly and immunocompromised individuals, as the VZV-specific cell-mediated immunity wanes, there exists a potential for reactivation of the latent virus. This reactivation, albeit rare, can lead to atypical manifestations, giving rise to unifocal or multifocal vasculopathy of the cerebral vasculature [2]. We report the clinical presentation, management, and outcomes of a patient admitted with VZV vasculopathy.

## Case presentation

A male 61-year-old manual labourer who had migrated from Northeast India to South India (Kerala) for work with no known comorbidities presented with loss of vision, ptosis of the left eye, weakness on the right side of his body, and deviation of the angle of his mouth to the left. He reported a history of vesicular eruptions over his left forehead and eyelid one month prior.

Upon examination, post-inflammatory scarring was noted over the dermatome supplied by the ophthalmic branch of the trigeminal nerve on the left side, suggestive of healed herpes zoster. The central nervous system examination revealed an obtunded patient with complete left ophthalmoplegia, right upper motor neuron (UMN) type facial palsy, and right hemiparesis. Additionally, signs of meningeal irritation were present.

**Figure 1:**
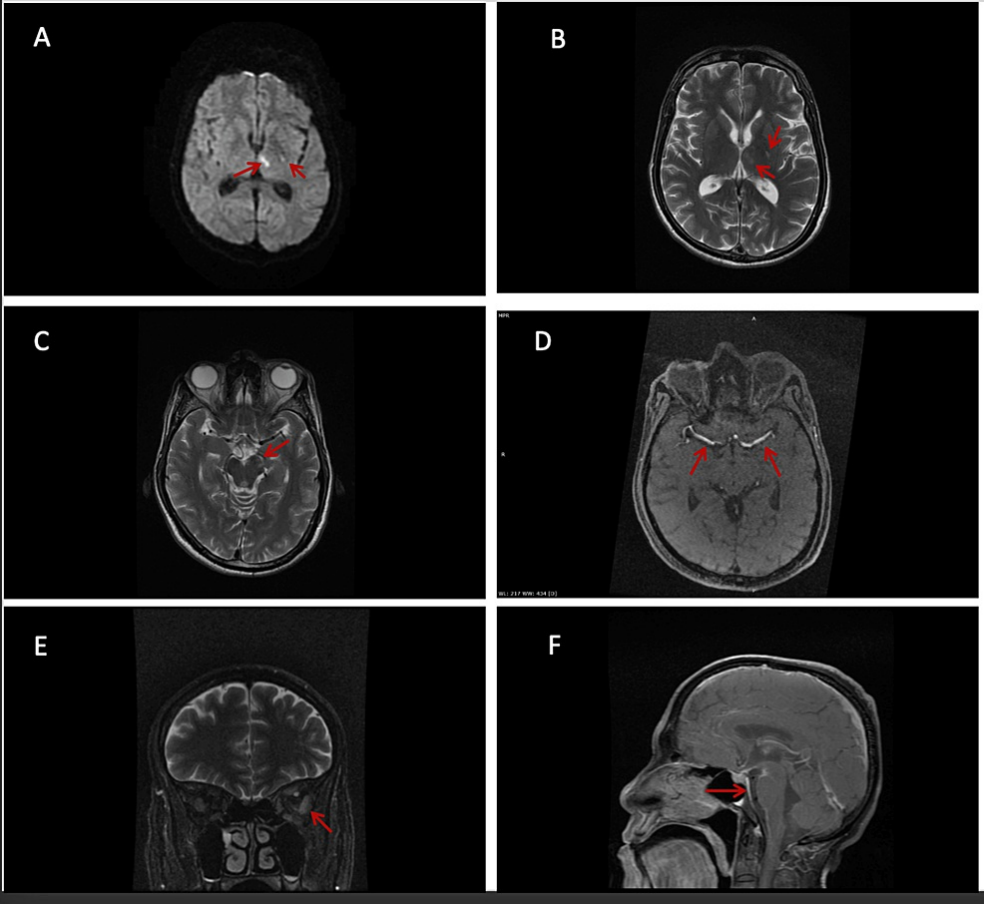
MRI of the Orbit and Brain With Angiography (A) T2 FLAIR image showing hyperintensity in the left thalamus and capsuloganglionic region (red arrows). (B) T2-weighted image showing hyperintensity in the left thalamus and capsuloganglionic region (red arrows). (C) T2-weighted image showing subtle hyperintensity in the left cerebellar peduncle of the midbrain (red arrow). (D) T1-weighted post-gadolinium axial image showing thickening and enhancement of the walls of the bilateral middle cerebral arteries (red arrows). (E) T2-weighted coronal image showing bulky left lateral rectus muscle (red arrow). (F) T1 post-gadolinium sagittal image showing abnormal prepontine leptomeningeal enhancement (red arrow).

The MRI of the orbit and brain with angiography revealed an acute infarct in the left capsuloganglionic region, accompanied by a chronic infarct in the corresponding area on the right (Figure 1A). Additionally, post-gadolinium imaging exhibited pronounced thickening and enhancement of the walls of the left middle cerebral artery (Figure 1B). Notably, there was also evidence of diffuse prepontine leptomeningeal enhancement.

A comprehensive blood workup indicated no evidence of the patient's immunocompromised status. A subsequent lumbar puncture and cerebrospinal fluid (CSF) analysis revealed an elevated white blood cell count with a predominance of lymphocytes (total count: 32 cells/mm, lymphocytes: 98%, neutrophils: 2%). Glucose levels were within the normal range at 87 mg/dL, while protein levels were elevated at 98 mg/dL. Polymerase chain reaction (PCR) testing yielded a positive result for VZV DNA.

Based on these findings, a diagnosis of varicella zoster vasculopathy with middle circulation stroke and meningoencephalitis accompanied by zoster ophthalmicus was established.

The patient was promptly initiated on a 14-day course of intravenous acyclovir, complemented by a five-day regimen of oral prednisolone at a dosage of 1mg/kg. Encouragingly, there was notable improvement observed in the patient's sensorium. Early implementation of physiotherapy further yielded positive results, notably in gait and facial palsy. However, despite these advancements, complete ophthalmoplegia and loss of vision persisted in the left eye.

Unfortunately, after initial treatment, the patient was lost to follow-up, hindering the assessment of long-term progress and potential interventions.

## Discussion

Varicella zoster vasculopathy can manifest in various clinical presentations, including ischemic and hemorrhagic strokes, giant cell arteritis, arterial aneurysms or dissection, cranial neuropathies, spinal cord infarcts, extracranial vasculopathy, venous sinus issues, and peripheral venous thromboses [2,3]. The pathogenesis of vasculitis syndromes is attributed to the trans-axonal spread of the virus to the surrounding arteries and veins. This leads to a viral infection of the arteries, resulting in the isolation of multinucleate giant cells, Cowdry bodies, and viral particles. Consequently, this process causes vascular remodelling, ultimately leading to thrombosis, dissection, and aneurysm formation [1,4,5]. In a retrospective study conducted over 10 years among patients admitted with acute stroke in Germany, the incidence of VZV vasculitis was found to be 0.05% [6]. The hazard ratio for the risk of stroke among patients infected with VZV was 1.31, particularly elevated in cases where zoster ophthalmicus was present [7,8]. VZV infection can also result in a twofold increase in the incidence of recurrent ischemic strokes and transient ischemic attacks among children [9].

The diagnosis of VZV vasculopathy relies on a combination of clinical suspicion, cerebrospinal fluid (CSF) findings, and imaging studies. According to Nagel and colleagues, 63% of patients with VZV vasculopathy exhibited a rash, 67% had CSF pleocytosis, and 70% of cases had four-vessel angiography abnormalities, while CT/MRI scans detected abnormalities in 97% of patients [10]. Angiography (MRA/CT) demonstrates segmental constrictions and post-stenotic dilation, occlusions, or even aneurysms. High-resolution vessel wall MRIs have unveiled vessel wall enhancement with diffuse irregularities affecting small and large vessels. In some cases, it may also manifest as moyamoya syndrome, particularly in children [11]. Imaging typically reveals infarcts occurring in single or multiple vascular territories, often exhibiting a potential correlation with skin lesions. Deep-seated infarcts tend to be more prevalent and are typically characterized by an oval and well-defined shape. Notably, infarcts at the grey-white matter junctions are considered a distinctive hallmark of VZV vasculitis. Thus comprehensive imaging analysis is a crucial diagnostic tool for identifying and characterizing VZV vasculopathy, providing valuable insights for accurate clinical management and treatment [1].

For virological confirmation in cerebrospinal fluid (CSF), the identification of intrathecal anti-VZV IgG antibodies proved to be more sensitive, with positive results observed in 93% of patients. In contrast, the detection of VZV DNA using PCR yielded positive results in only 30% of cases (p<0.001). It is important to note that if both tests yield negative results, one can definitively exclude the diagnosis of VZV vasculopathy [12]. Nagel and colleagues have shown variations in clinical and laboratory features between immunocompromised and immunocompetent patients. While rash and anti-VZV antibodies were more prevalent in the immunocompetent population, immunocompromised individuals exhibited higher instances of VZV DNA and CSF pleocytosis. Notably, the distinction in the isolation of VZV DNA was the only finding that achieved statistical significance [10].

The administration of oral antiviral therapy has been shown to reduce the incidence of stroke following a zoster infection [8]. Once vasculitis develops, the mainstay of treatment is intravenous acyclovir administered for 14 days at a dosage of 10-15mg/kg, administered every eight hours. In cases where patients exhibit a poor response to initial therapy, the treatment duration may be extended up to four weeks. For immunocompromised individuals, particularly those with HIV infection, it is advisable to consider a diligent approach by continuing valaciclovir at a dosage of 1g thrice daily for a period of up to three months [13]. The role of corticosteroids in the treatment of VZV vasculopathy is not well documented. However, it is important to consider their potential benefits. Corticosteroids possess both anti-inflammatory and immunomodulatory effects, which may serve to enhance the body's inflammatory response to virus-infected arteries. This principle aligns with the approach used in managing other infectious vasculitides such as tuberculous meningitis, neurocysticercosis, and neuroborreliosis. Therefore, exploring the use of corticosteroids in the context of VZV vasculopathy warrants further investigation [14].

Is there a role for vaccinating patients above 60 years and those who are immunocompromised? The Shingles Prevention Study demonstrated a significant reduction in postherpetic neuralgia and decreased morbidity associated with herpes zoster [13]. Based on the principle that vaccinated individuals exhibit higher VZV-specific cell-mediated immunity, a decline of which serves as the primary trigger for reactivation, it is essential to consider vaccination as a preventive measure. It is important to note that immunocompromised individuals were excluded from this study, thus limiting the generalizability of the results.

Benson et al. recently evaluated the efficacy of the zoster vaccine in HIV-infected individuals with CD4 counts greater than 200. Their findings demonstrated satisfactory immunogenicity, suggesting the potential benefits of vaccination in this specific population. Moreover, similar levels of immunogenicity were observed in patients with CD4 counts ranging from 50 to 199 cells/µL. However, the sample size in this subgroup was too small to draw definitive conclusions.

These observations highlight the potential advantages of extending vaccination efforts to immunocompromised individuals, particularly those living with HIV, and suggest that it may be a prudent strategy to enhance their immune response against VZV [15]. This aspect holds potential significance and warrants further investigation.

## Conclusions

VZV vasculopathy is a rare and devastating neurological syndrome, particularly impactful in immunocompromised individuals. Early recognition and timely administration of antivirals, along with adjunctive treatment, have the potential to enhance outcomes for those affected by VZV vasculopathy. Nevertheless, it remains an under-recognized phenomenon, with patients frequently experiencing poor prognoses.
